# From Beat to Risk: How Heart Rate Variability Predicts Arrhythmias in Type 2 Diabetes

**DOI:** 10.3390/life16030520

**Published:** 2026-03-21

**Authors:** Amelian Madalin Bobu, Ștefania-Teodora Duca, Andrei Ionut Cucu, Diana Alina Avieriței, Cosmina-Georgiana Ponor, Maria-Ruxandra Cepoi, Sandu Cucută, Bianca-Ana Dmour, Claudia Florida Costea, Gina Botnariu, Irina-Iuliana Costache-Enache

**Affiliations:** 1 Faculty of Medicine, Grigore T. Popa University of Medicine and Pharmacy Iași, 700115 Iasi, Romania; amelian.bobu@gmail.com (A.M.B.); cosminageorgiana.ponor@d.umfiasi.ro (C.-G.P.); cepoi_maria-ruxandra@d.umfiasi.ro (M.-R.C.); cucuta.sandu@d.umfiasi.ro (S.C.); ana.gherasim-dmour@d.umfiasi.ro (B.-A.D.); claudia.costea@umfiasi.ro (C.F.C.); ginabotnariu66@gmail.com (G.B.); irina.costache@umfiasi.ro (I.-I.C.-E.); 2 Cardiology Clinic, “St. Spiridon” County Emergency Clinical Hospital, 700111 Iasi, Romania; avieritei_diana@yahoo.com; 3 Faculty of Medicine and Biological Sciences, University Stefan cel Mare of Suceava, 720229 Suceava, Romania; andrei.cucu@usm.ro; 4 Department of Diabetes and Metabolic Diseases, Clinical Emergency Hospital St. Spiridon, 700111 Iasi, Romania

**Keywords:** heart rate variability, cardiac autonomic neuropathy, arrhythmias, arrhythmic predictability, RR interval variability, type 2 diabetes mellitus

## Abstract

Type 2 diabetes mellitus is associated with major cardiovascular complications, including cardiac autonomic neuropathy, which contributes to sympathetic–parasympathetic imbalance and increases susceptibility to arrhythmias and sudden cardiac death. Heart rate variability, assessed through R–R intervals on electrocardiography and 24 h Holter monitoring, represents a sensitive, non-invasive marker of autonomic dysfunction and arrhythmogenic risk. In patients with type 2 diabetes mellitus, chronic hyperglycaemia, oxidative stress, and metabolic inflammation lead to early impairment of the autonomic nervous system, manifested by consistent reductions in SDNN, RMSSD, pNN50, total power, and the high-frequency component, indicating diminished parasympathetic tone and sympathetic predominance. Nonlinear HRV indices demonstrate a loss of complexity and fractal organisation, providing additional prognostic value beyond conventional time- and frequency-domain analyses. Reduced HRV correlates with the severity of cardiac autonomic neuropathy, duration of diabetes, and poor glycaemic control, identifying patients with increased arrhythmogenic vulnerability. HRV analysis enables prediction of arrhythmic risk, facilitating the identification of high-risk individuals and guiding personalised interventions. The integration of HRV assessment into routine clinical practice may improve the early detection of subclinical autonomic neuropathy and optimise cardiovascular risk stratification and management in patients with type 2 diabetes mellitus.

## 1. Introduction

Diabetes mellitus is considered an important economic and social problem due to its long-term complications, such as premature death. According to estimates from the Diabetes Atlas, in 2024 approximately 589 million adults aged 20–79 years were living with diabetes, representing 11.1% of the global population within this age range. Of these, it is estimated that 252 million adults have undiagnosed diabetes [1].

In 2024, over 3.4 million people died due to diabetes, corresponding to 9.3% of total global deaths. Additionally, it is estimated that 9.1 million people live with type 1 diabetes, the majority (69%) being aged 20–59 years. Moreover, one in five births is affected by some form of hyperglycaemia occurring during pregnancy [1]. The number of people with diabetes is projected to continue rising in the coming decades. Projections indicate that the highest relative increase in prevalence will be observed in middle-income regions (13.5%), compared to 12.0% in high-income regions and 8.2% in low-income regions. At the same time, the most significant absolute increase is expected in these middle-income countries due to their large populations, particularly in countries such as China and India [2,3,4]. In Europe, an increase in diabetes incidence of approximately 10% is anticipated by 2050. By 2050, it is estimated that approximately 853 million adults will be living with diabetes globally [1]. The upward trend in diabetes prevalence is also reflected in the global geographic distribution. In 2024, according to the IDF Diabetes Atlas, the highest age-adjusted prevalence rates were reported in the Middle East and North Africa (19.9%), followed by North America and the Caribbean (13.8%) and the Western Pacific (11.1%) [1]. Regional disparities are considerable: while in some Middle Eastern countries, such as Pakistan (31.4%), prevalence exceeds 20%, in other regions, such as Western Europe, levels remain significantly lower, for example, Ireland (3.6%) [4].

These data highlight a significant increase in the incidence and impact of type 2 diabetes (T2DM) worldwide, with major implications for public health and mortality.

The aim of this narrative review is to summarise current evidence regarding cardiac autonomic neuropathy, heart rate variability, and arrhythmic risk in patients with type 2 diabetes mellitus.

## 2. Literature Search Strategy

This narrative review was conducted through a structured search of the scientific literature to identify studies addressing cardiac autonomic neuropathy, heart rate variability, and arrhythmic risk in diabetes mellitus, with a primary focus on type 2 diabetes mellitus. Electronic databases including PubMed, Scopus, and Web of Science were searched for articles published up to December 2025. The search strategy included combinations of the following keywords: “type 2 diabetes mellitus”, “diabetes mellitus”, “cardiac autonomic neuropathy”, “heart rate variability”, “arrhythmia”, “sudden cardiac death”, and “autonomic dysfunction”. Only English-language articles involving human subjects were considered. Original research articles, meta-analyses, narrative reviews, and guideline or consensus documents were included when relevant to the relationship between diabetes, autonomic dysfunction, HRV, and arrhythmic risk. Studies conducted on paediatric populations or animal models were excluded. Studies focusing primarily on type 1 diabetes mellitus were generally excluded, although seminal articles, consensus statements, and mechanistic studies including both type 1 and type 2 diabetes were retained when relevant for definitions or pathophysiological background. Priority was given to large cohort studies, meta-analyses, and guideline documents, while smaller mechanistic or experimental studies were included when necessary to support the pathophysiological mechanisms of autonomic dysfunction and arrhythmogenesis in diabetes. Given the narrative nature of this review, a formal risk-of-bias assessment using standardised tools was not performed.

## 3. Complications in Diabetes

Diabetes mellitus is a systemic metabolic disorder characterised by persistent hyperglycaemia, leading to progressive damage of multiple organs and systems, including the cardiovascular system, nervous system, kidneys, retina, and peripheral circulation, as a result of the complex interplay between metabolic and vascular mechanisms [5]. The major impact of diabetes on overall health status is primarily driven by its vascular complications, which are classified into two distinct categories: macrovascular complications, including coronary artery disease, cerebrovascular disease, and peripheral arterial disease, and microvascular complications, represented by diabetic kidney disease, diabetic retinopathy, diabetic neuropathy, and diabetic cardiomyopathy [6,7].

Cardiovascular complications constitute the leading cause of morbidity and mortality among patients with diabetes [8]. In this context, diabetic neuropathy—particularly its cardiac autonomic form, is a frequently underdiagnosed microvascular complication [9,10] with significant prognostic implications, as it is associated with cardiovascular autonomic dysfunction, the development of arrhythmias, and an increased risk of sudden cardiac death [9].

## 4. Cardiac Autonomic Neuropathy

Cardiac autonomic neuropathy (CAN) is defined, according to the Toronto Consensus Panel Subcommittee on Diabetic Neuropathy, as impairment of cardiovascular autonomic control in patients with confirmed diabetes mellitus, after the exclusion of other potential causes [11]. Data from the literature indicate an extremely variable prevalence, ranging from 2 to 91% in patients with type 1 diabetes mellitus and from 25 to 75% in those with type 2 diabetes mellitus, with variations depending on diabetes duration, diagnostic criteria, and the characteristics of the studied population [12,13]. A recent study published by Davis et al. demonstrated that, among patients with type 2 diabetes mellitus, possible cardiac autonomic neuropathy is present in 33.7% of cases, while definite forms are identified in 15.3% [14].

Despite being considered one of the most common complications of diabetes, CAN remains underreported, primarily due to the absence of simple and easily implementable population-based screening methods, as well as the time required for comprehensive assessment of cardiovascular autonomic function [12].

CAN shows significant correlations with age, duration of diabetes, and poor glycaemic control, and is frequently associated with other microvascular and macrovascular complications, including distal symmetrical polyneuropathy [15,16]. Moreover, factors such as obesity, smoking, and type 2 diabetes contribute to reduced heart rate variability, an essential marker of autonomic dysfunction [13,17].

Cardiac autonomic neuropathy was first described in 1975 by Low et al., who demonstrated that diabetic neuropathy also affects the autonomic control of the cardiovascular system [18]. This impairment results in an imbalance between the sympathetic and parasympathetic components, with early involvement of the parasympathetic fibres, particularly the vagus nerve, the principal parasympathetic nerve innervating the myocardium, which accounts for approximately 75% of all parasympathetic fibres [19,20]. The distal fibres of the vagus nerve are affected first, in a pattern similar to length-dependent diabetic peripheral neuropathy [21].

In the early stages of cardiac autonomic neuropathy, a reduction in vagally mediated HRV indices is often observed, together with resting tachycardia and impaired autonomic regulation [22]. Autonomic dysfunction develops early, increasing cardiovascular risk even before definite CAN becomes clinically established, and is further exacerbated by insulin resistance [23].

As the disease progresses, parasympathetic dysfunction leads to resting tachycardia and loss of the circadian rhythm of blood pressure, while subsequent sympathetic impairment results in orthostatic hypotension. In advanced stages of CAN, both parasympathetic and sympathetic components are severely affected, and the heart becomes functionally denervated, with a fixed resting heart rate [20,24]. These alterations may evolve towards the development of cardiac arrhythmias, often perceived by patients as palpitations, but which may also remain asymptomatic, significantly contributing to the overall increase in cardiovascular risk [13,20].

## 5. Cardiac Rhythm Disturbances and Arrhythmogenesis in Diabetes

Beyond the classic cardiovascular complications of diabetes mellitus, such as coronary artery disease and heart failure, an increased susceptibility to cardiac arrhythmias is increasingly being recognised [25]. Although the association between type 2 diabetes mellitus and coronary artery disease or ischaemic cardiomyopathy is well documented, its impact on the cardiac conduction system remains insufficiently explored, despite its growing clinical relevance [26].

In patients with diabetes mellitus, disease-specific factors such as chronic hyperglycaemia, systemic inflammation, and oxidative stress promote the development of diffuse myocardial fibrosis affecting the atria, ventricles, and the cardiac conduction system, thereby increasing susceptibility to cardiac arrhythmias [27]. In association with cardiovascular autonomic neuropathy and the electrolyte disturbances frequently encountered in type 2 diabetes mellitus, this pathological milieu generates an electrically unstable substrate, predisposing to a broad spectrum of arrhythmias, ranging from atrial fibrillation to malignant ventricular tachyarrhythmias [25,28].

Arrhythmic risk is further accentuated in diabetic patients with cardiac autonomic neuropathy. Previous data have demonstrated an increased nocturnal QT/RR ratio in this population, correlated with altered ventricular repolarisation and a higher incidence of ventricular arrhythmias compared with patients without CAN [29,30]. In this context, autonomic imbalance between sympathetic and parasympathetic neurotransmitters, together with abnormal norepinephrine signalling and metabolism, may explain the increased incidence of cardiac arrhythmias and sudden cardiac death [31].

### Electrical Remodelling

In addition to mechanical alterations, diabetes also induces changes in cardiac electrical function, frequently manifested by electrical remodelling and prolongation of ventricular repolarisation, in both type 1 and type 2 diabetes mellitus [25,32]. Hyperglycaemia increases the production of reactive oxygen species (ROS), leading to dysfunction of HERG (human ether-à-go-go-related gene) ion channels. These channels represent essential subunits of the rapid delayed rectifier potassium current (IKr), and their impairment results in prolonged ventricular repolarisation and an increased risk of ventricular arrhythmias, including torsades de pointes [33,34].

Concomitantly, hyperglycaemia promotes the formation of advanced glycation end products (AGEs), which activate RAGE (receptor for advanced glycation end products) and stimulate NADPH oxidase, further amplifying ROS production [35]. Excess ROS interferes with ion channel function, disrupts intracellular calcium homeostasis via protein kinase C (PKC) activation, and favours the occurrence of delayed afterdepolarisations. Acute hyperglycaemia also increases inducible nitric oxide synthase (iNOS) expression, promoting excessive nitric oxide production and peroxynitrite formation, reactive species that induce mitochondrial dysfunction and alter excitation–contraction coupling [36,37,38].

**Figure 1:**
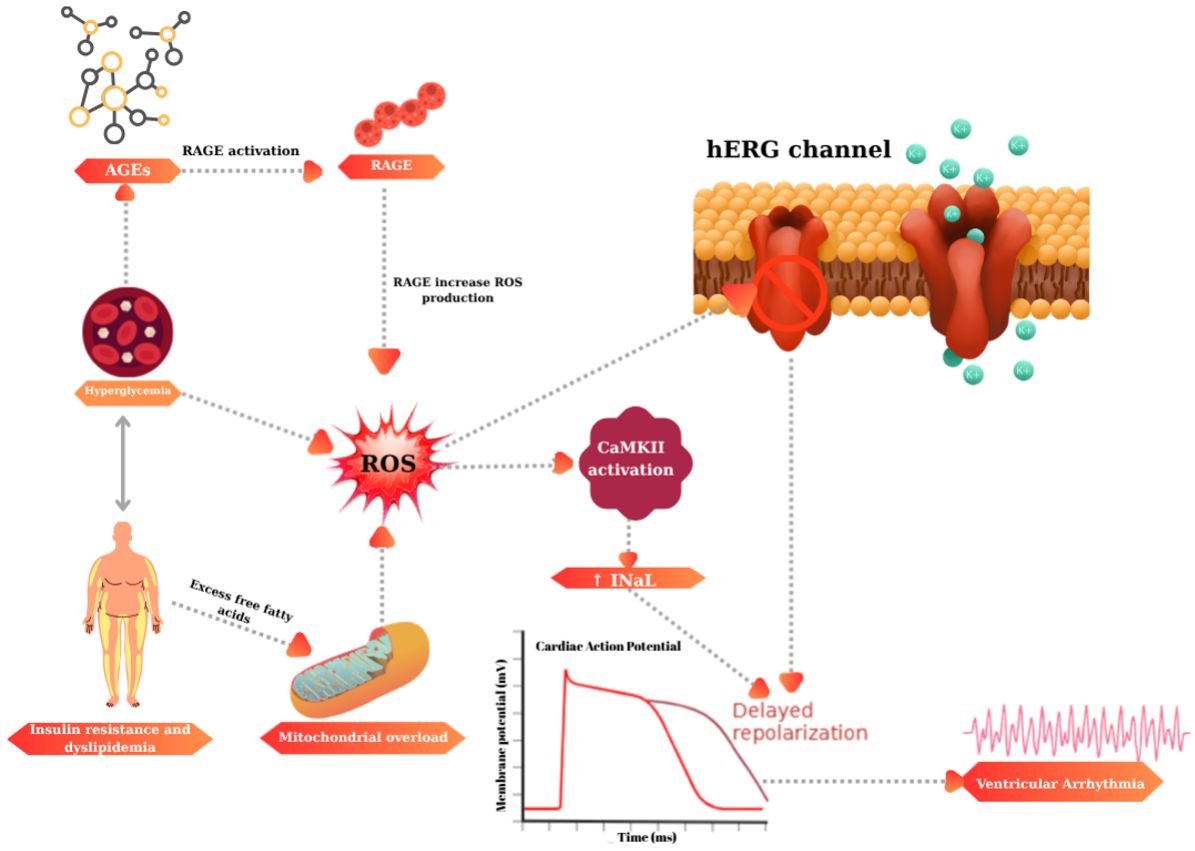
The Hyperglycaemia–ROS–Ion Channel Axis in Ventricular Arrhythmic Vulnerability. AGEs—advanced glycation end products; RAGE—receptor for advanced glycation end products; ROS—reactive oxygen species; hERG—human ether-à-go-go-related gene; IKr—rapid delayed rectifier potassium current; CaMKII—Ca^2+^/calmodulin-dependent protein kinase II; INaL—late sodium current; PKC—protein kinase C; iNOS—inducible nitric oxide synthase. ↑—Up.

In addition, hyperglycaemia and oxidative stress may promote activation of Ca^2+^/calmodulin-dependent protein kinase II (CaMKII), a key regulator of cardiac ion channels and intracellular calcium handling. CaMKII activation has been shown to enhance the late sodium current (INaL), which contributes to intracellular sodium loading, prolongation of the action potential duration (APD), and increased electrical instability. These mechanisms may further facilitate ventricular arrhythmogenesis in the diabetic myocardium [39,40]. These processes are summarised in Figure 1.

In the context of insulin resistance and hyperlipidaemia associated with diabetes, mitochondrial overload due to excess fatty acids further enhances ROS generation, impairing intercellular coupling and slowing electrical conduction [41,42].

## 6. Cardiac Autonomic Neuropathy: From Pathophysiology to Clinical Detection Through RR Interval Variability

### 6.1. HRV Parameters and Methods of Assessment

Cardiac autonomic dysfunction was first recognised in 1965, when Hon and Lee reported the clinical significance of HRV [43]. Since then, studies have demonstrated that autonomic neuropathy develops progressively in diabetes, even before the onset of overt clinical symptoms, reflecting an imbalance between the sympathetic and parasympathetic branches of the autonomic nervous system [15,44,45,46,47,48,49].

HRV, measured using R–R intervals on electrocardiography, has become the gold standard for the assessment of cardiac autonomic neuropathy. Precise determination of these intervals is essential for quantifying time-domain and frequency-domain changes, as well as nonlinear parameters such as SD1/SD2 and fractal analysis, which are relevant for cardiovascular risk stratification [50,51,52,53,54].

**Figure 2:**
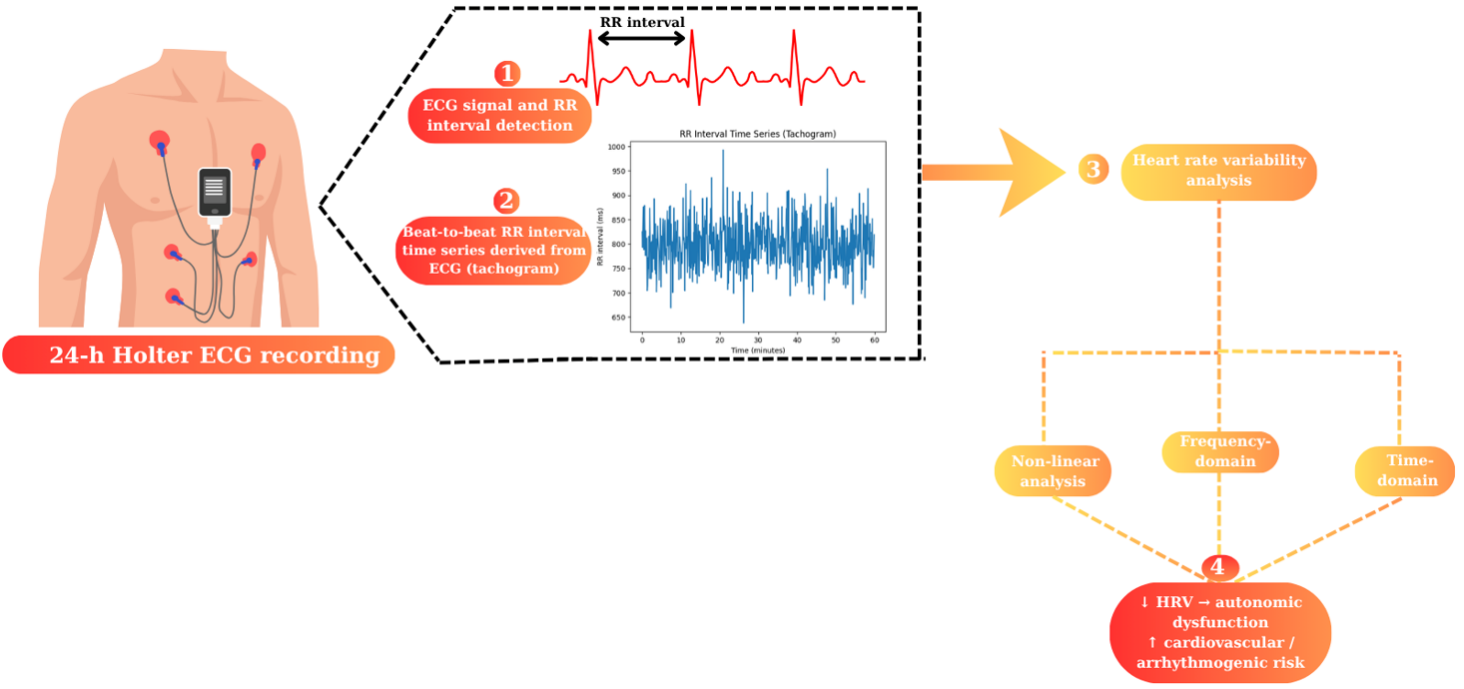
Arrhythmic risk stratification based on HRV analysis. ↑—Up; ↓—Down.

R–R interval variability can be quantified using a variety of electrocardiographic methods, each with specific clinical utility. A standard 10 s ECG allows rapid screening, while recordings of approximately 5 min are used for frequency-domain HRV analysis. However, 24 h Holter ECG monitoring remains the gold standard, as it enables assessment of circadian variability, dynamic autonomic responses, and associated arrhythmic episodes (Figure 2) [22,55].

In patients with diabetes, global HRV parameters such as SDNN and total power (TP) progressively decline, indicating loss of the heart’s ability to adapt to daily physiological demands and simultaneous impairment of both sympathetic and parasympathetic components. Short-term parameters, RMSSD, pNN50, and SD1, end to decrease earlier, reflecting loss of parasympathetic tone. The HF component, correlated with respiration-dependent vagal activity, is consistently reduced, while the LF component often decreases in parallel, suggesting global depression of autonomic modulation. Poincaré plot analysis often demonstrates reduced SD1 and altered SD1/SD2 ratio, consistent with impaired short-term beat-to-beat variability and altered autonomic modulation. Reduction in the VLF band is particularly relevant, as it represents an independent predictor of cardiovascular mortality [22,44,46,56,57].

#### 6.1.1. Methodological Limitations in HRV Interpretation

However, the physiological interpretation of HRV indices requires important methodological caution. Traditionally, frequency-domain parameters, particularly the LF/HF ratio, have been interpreted as markers of “sympathovagal balance,” with higher values assumed to reflect sympathetic predominance and lower values parasympathetic predominance. Within this framework, LF power has often been regarded as a surrogate of sympathetic modulation, whereas HF power has been considered an index of vagal activity. This interpretation has increasingly been challenged. Experimental and physiological evidence indicates that the LF component does not represent a specific marker of cardiac sympathetic activity but rather reflects a complex interaction among sympathetic and parasympathetic influences, baroreflex mechanisms, respiratory dynamics, and other physiological processes. For example, studies using pharmacological autonomic blockade and selective denervation have demonstrated that parasympathetic inhibition markedly reduces LF power, indicating a substantial vagal contribution to this frequency band [58,59], while direct recordings of sympathetic nerve activity have shown weak or absent correlations with LF variability [60,61]. In addition, physiological conditions associated with increased sympathetic activation, such as exercise or myocardial ischemia, do not consistently increase LF power and may even reduce it [62]. Based on this body of evidence, Billman demonstrated that the LF/HF ratio does not reliably quantify cardiac sympathovagal balance and that interpreting this metric as a direct index of sympathetic–parasympathetic antagonism oversimplifies the nonlinear interactions that characterise autonomic cardiovascular regulation [63].

Importantly, these conceptual limitations are frequently overlooked in clinical and epidemiological studies, where LF, HF, and particularly the LF/HF ratio are often interpreted as direct markers of autonomic balance.

An additional limitation arises from the intrinsic mathematical dependency of many HRV indices on mean heart rate. Time-domain measures (e.g., SDNN, RMSSD) and absolute spectral powers (LF, HF, total power) are directly linked to mean cycle length, such that higher heart rates reduce HRV while slower rates increase R–R variability even without changes in autonomic modulation. Consequently, HRV indices cannot be interpreted reliably as markers of autonomic balance when baseline heart rate differs between conditions. Early experimental evidence for this concept was provided by Rocchetti et al., who showed in isolated sinoatrial myocytes that cholinergic slowing of pacemaker activity increases cycle-length variability through changes in the rate of diastolic depolarization, indicating that HRV depends not only on neural fluctuations but also on the tonic level of autonomic input and the intrinsic input–output properties of the sinoatrial pacemaker [64]. Zaza and Lombardi subsequently developed this concept further, proposing that autonomic indices derived from sinus cycle length, including HRV parameters and baroreflex sensitivity, display intrinsic rate dependency because the relationship between neural input and sinus node response is nonlinear [65]. Monfredi et al. later independently confirmed and broadened this concept by demonstrating a consistent inverse relationship between HRV and mean heart rate across species and experimental conditions, indicating that part of HRV variability reflects intrinsic electrophysiological properties of sinoatrial pacemaking rather than autonomic input alone [66].

Consistent with these observations, pharmacological and modelling studies further highlight the influence of heart rate on HRV metrics. Selective heart-rate-lowering interventions, such as ivabradine, have been shown to modify HRV indices independently of direct changes in autonomic neural activity [67]. In parallel, mathematical and signal-processing analyses indicate that both spectral and complexity-based HRV measures are highly sensitive to mean heart rate and may therefore distort estimates of autonomic modulation when heart rate differs across physiological or pathological states [68].

Importantly, these methodological considerations are often insufficiently addressed in clinical and epidemiological studies, where HRV indices are frequently interpreted as direct markers of autonomic balance without accounting for baseline heart rate. Consequently, part of the heterogeneity reported in the literature may reflect differences in intrinsic sinus node dynamics rather than true variations in autonomic regulation. HRV should therefore be interpreted not as a simple surrogate of autonomic outflow but as a composite physiological signal shaped by autonomic modulation, sinus node transduction properties, respiratory influences, and prevailing heart rate.

#### 6.1.2. Clinical and Epidemiological Evidence Linking HRV and Diabetes

Population-based studies suggest that cardiac autonomic dysfunction may occur early in the course of metabolic dysregulation, sometimes preceding the clinical onset of diabetes mellitus. For example, analysis of the Tromsø 6 cohort demonstrated significant reductions in HRV indices, particularly SDNN and RMSSD, among individuals with metabolic syndrome and diabetes, including those below the diagnostic threshold for diabetes. In this cohort, SDNN decreased progressively with an increasing number of metabolic syndrome components and higher HbA1c levels, suggesting a dose–response relationship between metabolic burden and impairment of autonomic regulation [69]. However, these findings should be interpreted with caution, as HRV was estimated from pulse-derived variability obtained from ultra-short recordings, and the cross-sectional design does not allow causal inference. Prospective cohort studies provide additional evidence supporting this association. In a large Asian longitudinal cohort including more than 54,000 adults, reduced vagal activity, reflected by lower SDNN, RMSSD, and normalised HF power—was associated with an increased risk of incident diabetes during follow-up. Higher resting heart rate and changes in several frequency-domain HRV parameters were also linked to the subsequent development of diabetes, independent of traditional cardiometabolic risk factors [70]. Given the ongoing methodological debate regarding the interpretation of spectral HRV measures, these findings are more appropriately interpreted as reflecting alterations in autonomic dynamics rather than a direct quantification of “sympathovagal balance.” Moreover, HRV was derived from relatively short recordings of approximately three minutes, which may reduce the stability of these estimates compared with longer ECG recordings.

Additional insights are provided by the Rotterdam Study, a large population-based prospective cohort in which HRV measurements were repeatedly assessed over time. In this investigation, higher resting heart rate and lower heart-rate-corrected RMSSD were independently associated with the development of type 2 diabetes during a median follow-up of 8.6 years, particularly among younger individuals. An important methodological strength of this study lies in the use of repeated HRV measurements combined with longitudinal statistical modelling, allowing autonomic parameters to be evaluated as dynamic exposures over time. However, Mendelian randomization analyses did not support a direct causal relationship between HRV and diabetes, suggesting that autonomic dysfunction may represent an early marker or mediator of metabolic dysregulation rather than a primary etiological factor [71].

Complementary clinical monitoring studies provide additional mechanistic insights into the relationship between glycaemic control and autonomic function. Investigations combining continuous glucose monitoring with ambulatory ECG recordings have shown that poorly controlled diabetes is associated with reductions in time-domain HRV parameters, including SDNN, RMSSD, NN50, and pNN50, as well as decreases in total spectral power and in VLF, LF, and HF components, suggesting a complex autonomic dysfunction involving impairment of both parasympathetic and sympathetic mechanisms [72]. Real-time analyses further indicate an inverse relationship between glycaemic levels and HRV, implying that acute hyperglycaemia may directly influence autonomic regulation. Consistent with these findings, persistent hyperglycaemia and poor glycaemic control have also been associated with significantly lower values of the global HRV marker SDNN and predominantly parasympathetic parameters—RMSSD, SDSD, NN50, and pNN50—during periods of inadequate metabolic control (*p* < 0.001), while frequency-domain analysis demonstrates reduced total power and decreased VLF, LF, and HF components. However, these studies generally involve relatively small clinical samples, which limits the generalizability of the findings.

### 6.2. Autonomic Dysfunction and Cardiovascular

Epidemiological studies and cohort analyses consistently suggest that cardiac autonomic dysfunction has important prognostic implications in patients with type 2 diabetes, although the level of evidence varies depending on study design and the methods used to assess HRV. In a large prospective cohort that included more than 8900 participants evaluated during metabolic screening, Kataoka et al. demonstrated that a marked reduction in R–R interval variability (CVR–R < 2.2%) was associated with approximately a twofold increased risk of sudden cardiac death in individuals with diabetes, even after adjustment for traditional cardiovascular risk factors. Interestingly, this association was not observed in the non-diabetic population, suggesting that autonomic dysfunction may play a particular role within the specific metabolic context of diabetes [73]. However, HRV assessment was based on very short ECG recordings, which may limit the accuracy of autonomic regulation estimation compared with longer-duration ECG monitoring.

Consistent findings were reported by Hillis et al. in an analysis of the ADVANCE cohort including 11,140 patients with type 2 diabetes mellitus. Higher resting heart rate was independently associated with increased risks of all-cause mortality, cardiovascular death, and major cardiovascular events, with each 10 beats per minute increase corresponding to an approximately 15% higher risk of death after adjustment for multiple covariates [74]. However, resting heart rate is an indirect marker of autonomic dysfunction and may reflect multiple physiological and clinical factors; thus, it should be interpreted primarily as a prognostic indicator rather than direct evidence of sympathovagal imbalance. Extending these observations to populations with elevated metabolic risk, the ADDITION-PRO (n = 1627), study demonstrated that multi-day and hourly HRV assessment provides additional insights into cardiovascular risk. In this cohort, low SDNN (<120 ms) indicated the highest risk of cardiovascular complications. Hourly analysis further revealed that reduced HRV in the morning (06:00–07:00 a.m.) was most strongly correlated with major cardiovascular events, suggesting impaired autonomic adaptation during the period of increased physiological activation after awakening. Similarly, elevated heart rate during the night (02:00–06:00 a.m.) was associated with higher risk of complications and mortality [75]. However, HRV estimates were derived from wearable heart-rate monitoring rather than standard ECG recordings, and the observational design in a high-risk cohort limits causal inference and generalizability.

These findings support the concept that HRV and heart rate reflect long-term autonomic adaptability and may serve as clinically relevant prognostic markers in populations at metabolic risk.

Another study by Kaze et al. showed that cardiac autonomic dysfunction in adults with type 2 diabetes, defined by very low HRV values (SDNN < 8.2 ms and RMSSD < 8.0 ms), was associated with an almost twofold increased risk of silent myocardial infarction [76]. However, HRV was assessed from 10 s ECG recordings and silent myocardial infarction was identified by ECG criteria alone, which may limit both physiological interpretation and event ascertainment. Consistent with the nocturnal pattern observed in the ADDITION-PRO cohort, the Copenhagen Holter Study found that reduced nocturnal HRV, measured as SDNN during sleep (02:00–02:15 a.m.), was associated with increased cardiovascular risk, and that each 10 ms increment in nocturnal SDNN was linked to a significant reduction in risk [77]. Although these findings suggest that nocturnal HRV may improve cardiovascular risk stratification, the relatively small diabetic subgroup and the predominantly European low-risk population limit broad generalizability.

As autonomic neuropathy progresses, HRV reduction correlates with disease severity and overall mortality. Both the studies by Whang et al. and the MONICA/KORA cohort indicated that diabetic patients with reduced HRV have a higher risk of arrhythmic death and rhythm instability, and that a prolonged QTc interval exceeding 440 ms serves as an independent predictor of mortality. Moreover, 24 h QT/RR dynamic analysis can provide an earlier indicator of autonomic impairment than classical HRV or QT parameters [29,78,79]. However, the studies differ substantially in design and measurement: Whang et al. evaluated post-myocardial infarction patients using 24 h Holter-derived HRV indices, whereas MONICA/KORA relied on short resting ECG recordings in a general elderly population. These methodological differences indicate that the observed associations likely reflect different aspects of autonomic and electrophysiological risk. Moreover, dynamic QT/RR analysis may detect autonomic impairment earlier than conventional HRV or static QT measures [78,79].

Cardiac autonomic neuropathy also constitutes an independent predictor of diabetic nephropathy progression and chronic kidney disease in patients with diabetes, highlighting its systemic impact and overall prognostic significance [15,80,81].

### 6.3. HRV and Arrhythmic Risk

Cardiac autonomic dysfunction in patients with diabetes is associated with an increased risk of both atrial fibrillation (AF) and ventricular arrhythmias. Imbalance between sympathetic and parasympathetic activity predisposes to the development of AF, an observation confirmed in both population-based studies and experimental models in diabetic rats, which exhibited a higher incidence of AF following sympathetic stimulation [82,83,84,85]. In humans, AF represents the most common arrhythmia among diabetic patients, with reported prevalences exceeding 70% [27].

HRV parameters have demonstrated predictive value in this context: patients who developed AF exhibited increased time-domain (ASDNN, RMSSD, pNN50, BB50) and frequency-domain (LF, HF) HRV measures, reflecting abnormal autonomic fluctuations preceding the onset of arrhythmia [86,87,88]. Multivariate analyses confirmed that the presence of any of these “at-risk” parameters was independently associated with AF occurrence (HR 2.396; *p* = 0.017) [86], while data from the ARIC study indicated that lower HRV values increase the long-term risk of developing atrial fibrillation [89].

Beyond AF, cardiac autonomic dysfunction significantly increases susceptibility to ventricular arrhythmias. Although less frequent in the general population of patients with type 2 diabetes, these arrhythmias carry major clinical relevance, particularly in individuals with ischaemic cardiomyopathy or a history of myocardial infarction. Ventricular tachycardia has been reported in approximately 4–17% of diabetic patients, while ventricular extrasystoles and other ventricular arrhythmias occur in 20–25% of individuals with diabetes and associated cardiovascular risk factors [27].

Data from ambulatory recordings suggest that heart rate variability changes precede the spontaneous onset of ventricular tachyarrhythmias, indicating that HRV abnormalities may predispose patients to arrhythmias or at least signal the presence of a transient trigger, although these observations derive from small, selected studies and remain partially controversial [90,91].

Evidence also suggests that short-term alterations in R–R interval dynamics may precede ventricular tachyarrhythmias. Rozen et al. evaluated a multipole-based HRV analysis approach in patients with implantable cardioverter-defibrillators and reported high specificity (91.6%) and overall predictive accuracy of approximately 84.5% for the detection of imminent ventricular tachyarrhythmias using a multipole-derived HRV parameter (Dyx), derived from Poincaré plot-based analysis of R–R interval dynamics and combined with transient heart rate acceleration [92]. Pathological HRV patterns were frequently detectable 15 to 60 min before the arrhythmic event, suggesting that dynamic changes in R–R interval organisation may precede ventricular tachyarrhythmias. However, the study was based on retrospective analysis of stored device recordings in a relatively small and highly selected cohort of 28 patients with implantable cardioverter-defibrillator (ICDs), which limits the generalizability of these findings.

More recently, machine learning approaches have been applied to HRV analysis to identify short-term patterns associated with imminent arrhythmic events. Ebrahimzadeh et al. evaluated combinations of time-domain, frequency-domain, time–frequency, and nonlinear HRV features, including SDNN, RMSSD, LF/HF ratio, and Poincaré-derived indices, and reported classification accuracies exceeding 80% for ventricular tachyarrhythmias occurring within the final minutes preceding sudden cardiac death [93]. Similarly, Kitlas-Golińska et al. applied Random Forest-based models to RR-interval signals from patients with implantable cardioverter-defibrillators, using a broad set of descriptors derived from time-domain, spectral, and nonlinear HRV analyses, achieving moderate predictive performance (AUC ≈ 0.82) for distinguishing normal rhythm from arrhythmia onset [94]. However, both studies relied on relatively small retrospective datasets and cross-validation procedures, which limit their direct clinical applicability. Moreover, within machine learning frameworks conventional HRV metrics, including spectral indices such as LF, HF, or the LF/HF ratio, function primarily as statistical features rather than physiologically specific markers of autonomic balance.

Taken together, these findings suggest that dynamic alterations in RR-interval organisation may precede malignant ventricular arrhythmias and can be captured by advanced analytical methods. However, current evidence indicates that HRV-based models primarily identify statistical patterns associated with arrhythmic events rather than providing direct mechanistic insight into autonomic regulation, underscoring the need for larger prospective studies to clarify their clinical utility.

Deep-learning approaches have also been explored for predicting imminent ventricular tachyarrhythmias from HRV signals. In a study using the PhysioNet spontaneous ventricular tachyarrhythmia database (78 ICD patients), a one-dimensional convolutional neural network achieved a prediction accuracy of 84.6% approximately 60 s before arrhythmia onset [93,95]; however, the relatively small dataset and reliance on data augmentation limit generalizability.

In type 2 diabetes mellitus, the severity of cardiac autonomic neuropathy appears to correlate closely with the occurrence of ventricular arrhythmias, particularly during the night. In a cross-sectional study of 219 hospitalised patients with type 2 diabetes conducted in China, Chen et al. showed that patients with nocturnal ventricular arrhythmias had significantly lower heart rate variability during deep breathing and more impaired postural blood pressure responses, suggesting concomitant abnormalities in both parasympathetic and sympathetic cardiovascular control. The incidence of nocturnal ventricular arrhythmias increased progressively with CAN severity, reaching 60% in patients with advanced CAN (Ptrend = 0.034), and multivariable analysis identified CAN stage as an independent correlate of these events (OR, 1.765; 95% CI, 1.184–2.632; *p* = 0.005) [30]. Notably, this association was observed specifically during the nocturnal period and not during daytime recordings, supporting the possibility that circadian changes in autonomic regulation may modulate arrhythmic vulnerability, a pattern also reported in the Copenhagen Holter Study, which identified reduced nocturnal HRV as a predictor of cardiovascular risk [77]. However, these findings should be interpreted with caution because the study was cross-sectional, included a relatively small subgroup with advanced CAN, and was conducted in a single-centre inpatient cohort in China, which limits causal inference and generalizability. Accordingly, the data support the association between nocturnal arrhythmic burden and autonomic dysfunction but do not establish that HRV measures directly quantify sympathovagal balance or independently predict sudden cardiac death during sleep.

In line with these observations, Whang et al. showed that diabetic patients surviving an acute myocardial infarction exhibit significantly higher arrhythmic mortality compared with non-diabetic patients (17% vs. 8%), accompanied by marked reductions in R–R interval variability. Global HRV parameters, including SDNN <50 ms, total power <2000 ms^2^, as well as ULF (<1600 ms^2^) and VLF (<180 ms^2^) components, independently predicted arrhythmic and overall mortality in diabetic patients, with predictive performance comparable to that observed in non-diabetic populations [78]. However, these findings should be interpreted with caution because the analysis was derived from a historical post–myocardial infarction cohort in which only a relatively small subgroup had diabetes (n = 117), and the data were collected before the widespread use of contemporary reperfusion strategies and modern cardioprotective therapies. Moreover, HRV parameters in this context likely reflect a combination of post-infarction autonomic remodelling, sinus node dynamics, and structural myocardial disease rather than a specific measure of cardiac autonomic neuropathy. Overall, these results nevertheless support the concept that impaired autonomic–cardiac regulation contributes to arrhythmogenic vulnerability and adverse cardiovascular prognosis in type 2 diabetes mellitus.

In a cross-sectional study of 411 patients with type 2 diabetes, Cha et al. demonstrated that prolonged QTc interval (>440 ms) is significantly associated with reduced HRV parameters in both time-domain (SDNN, RMSSD) and frequency-domain (LF, HF) measures. Patients with prolonged QTc had a 2–4-fold higher likelihood of reduced HRV indices (e.g., OR = 3.99 for TP and OR = 3.31 for SDNN), independent of age, sex, diabetes duration, and HbA1c. These findings support the link between cardiac autonomic dysfunction and arrhythmogenic vulnerability in type 2 diabetes [96], consistent with this association, earlier observations by Ewing et al. suggested that QTc interval prolongation parallels the severity and progression of autonomic dysfunction and was greater in diabetic individuals who subsequently died unexpectedly, possibly due to malignant ventricular arrhythmias [97]. However, the cross-sectional design of the study by Cha et al. precludes causal inference, while the study by Ewing et al. was retrospective and based on relatively small male-only cohorts. Accordingly, although these studies support a potential relationship between autonomic dysfunction, QT prolongation, and arrhythmic vulnerability, they do not establish QTc prolongation as a specific or validated prognostic marker of cardiac autonomic neuropathy.

Table 1 summarises the main HRV parameters in patients with type 2 diabetes, highlighting characteristic changes, clinical relevance, and prognostic significance.

The available literature is markedly heterogeneous with respect to ECG acquisition protocols, recording duration, HRV preprocessing, diabetes phenotype, glycaemic control, comorbid cardiovascular disease, and outcome definitions. These differences limit direct comparisons across studies and may partly explain inconsistent findings, particularly for frequency-domain and nonlinear HRV indices.

## 7. Clinical Implications and Future Directions: Toward Integrated Arrhythmic Risk Stratification in Diabetes

HRV analysis derived from short ECG recordings provides a rapid, non-invasive, and reliable method for detecting cardiac autonomic dysfunction in patients with diabetes, representing a promising tool for arrhythmic risk stratification and guidance of early clinical interventions [47]. Although CAN is often asymptomatic and may occur in subclinical stages, current recommendations from the American Diabetes Association (ADA) do not support routine screening for subclinical cardiovascular disease in the absence of symptoms or evident risk factors [98]. Nevertheless, recent evidence suggests that early identification of CAN may have prognostic value, given its association with increased risk of cardiovascular events and mortality in patients with diabetes [75,77,99].

In this context, 24 h HRV monitoring provides a more comprehensive and predictive assessment than short-term measurements. Deviations of HRV values from normal ranges correlate with various cardiovascular conditions, and 24 h SDNN values allow objective cardiac risk stratification, facilitating the identification of high-risk patients and guiding preventive and therapeutic strategies [100,101,102].

Early detection of CAN is essential, given that in its initial stages it can be partially reversible and respond favourably to lifestyle interventions [20,103]. Management of CAN should adopt a multifactorial approach, focusing on optimising glycaemic control, correcting dyslipidaemia, and appropriately treating hypertension. Early initiation of interventions is crucial, as CAN represents an independent prognostic factor for major cardiovascular events and mortality, emphasising the need to integrate autonomic function assessment into modern risk stratification strategies for patients with diabetes [12,15].

HRV monitoring allows early detection of cardiac autonomic dysfunction in patients with diabetes or prediabetes, identifying individuals at increased risk of arrhythmias. Lifestyle interventions, such as hypocaloric diets, regular physical exercise, or bariatric surgery [104,105], together with certain pharmacological treatments, including metformin or SGLT2 inhibitors, which can reduce excessive sympathetic activation and restore autonomic balance [106,107], increase HRV and reduce the risk of CAN, highlighting the preventive and reversible potential of this cardiovascular complication.

In this context, Anderson et al. demonstrated that cardiac autonomic neuropathy can be partially reversible when diagnosed at an early stage and intensive glycaemic control is implemented promptly, suggesting that early therapeutic intervention can modify the course of autonomic dysfunction [108]. HRV assessment can guide personalised treatment in patients with type 2 diabetes, as shown in the ACCORD study involving 7946 patients. Reduced HRV identified subgroups that benefited from intensive glycaemic control, with a reduction in cardiovascular events and no increase in mortality, whereas patients with normal HRV derived no benefit and faced a higher risk of adverse events [109].

A second study, conducted within the ACCORDION framework, included 7866 patients with type 2 diabetes, followed for approximately nine years after the completion of the ACCORD trial. This study complements and confirms the initial observations, showing that patients with CAN, objectively assessed using ECG-derived measures, derive significant benefits from intensive glycaemic therapy, with reductions in both cardiovascular events and all-cause mortality. In contrast, patients without CAN did not experience similar effects. Risk differences associated with the presence of CAN, evident in the standard treatment group, were attenuated by intensive therapy, demonstrating that intensifying glycaemic control can counteract the adverse impact of cardiac autonomic dysfunction [110]. However, these findings should be interpreted with caution. CAN was defined using HRV indices derived from a short 10 s resting ECG rather than from standard cardiovascular autonomic reflex tests or long-term HRV recordings, which may limit diagnostic precision. In addition, the present analysis represents a post hoc evaluation of the ACCORD/ACCORDION cohort rather than a trial specifically designed to test treatment effects according to CAN status. Consequently, although the results support the potential value of short-term HRV assessment for risk stratification and therapeutic guidance, prospective studies specifically targeting patients with confirmed CAN are needed to validate these observations.

HRV analysis has demonstrated that therapeutic interventions can modulate cardiac autonomic function and reduce arrhythmic and cardiovascular risk. For example, in studies of patients with HFpEF, beta-blocker therapy increased HRV parameters (RMSSD and HF power), suggesting recovery of parasympathetic tone and changes in autonomic markers, particularly during time periods associated with a higher risk of cardiovascular events [111,112,113,114,115]. However, these data derive largely from heterogeneous non-diabetic populations and do not establish that treatment-related HRV changes are themselves the mechanism of clinical benefit. HRV should therefore be interpreted primarily as a dynamic marker of physiological response rather than a validated surrogate of therapeutic efficacy.

## 8. Future Directions

Evaluation of heart rate variability represents a valuable method for early detection of cardiac autonomic neuropathy in patients with type 2 diabetes, even before the appearance of overt clinical symptoms. This approach may allow early identification of patients with subclinical autonomic dysfunction and, consequently, more precise cardiovascular risk stratification [116].

For HRV measurement to become truly clinically relevant in patients with diabetes, it is necessary to identify effective interventions for those with abnormal HRV values, which reflect cardiac autonomic dysfunction and arrhythmogenic vulnerability. Ongoing studies are investigating whether pharmacological or non-pharmacological interventions—including antiarrhythmic treatments, optimisation of glycaemic control, lifestyle modifications, or emerging therapies—can improve survival, prevent progression of autonomic neuropathy, and reduce the risk of major cardiovascular events. It is crucial to determine whether normalising HRV through these strategies can effectively lower mortality and arrhythmia incidence, thereby avoiding the historical pitfalls of interventions that failed to influence prognosis. In the long term, if HRV research in diabetes continues at the accelerated pace seen over the past decades, it is plausible that measurement of a simple HRV index could become a routine procedure in diabetes care, comparable to blood pressure or HbA1c assessment, providing a practical tool for cardiovascular risk stratification and guiding personalised interventions [80,117,118,119].

Beyond its role in the early detection of cardiac autonomic neuropathy and cardiovascular risk stratification in patients with diabetes, recent research has explored the use of artificial intelligence for predicting type 2 diabetes and associated cardiovascular events. Machine learning models such as Random Forest, LightGBM, and CatBoost have identified HRV parameters from time-domain, frequency-domain, and nonlinear analyses (e.g., SD2, SDRR, CVRR) as strong predictors of autonomic dysfunction, demonstrating the clinical utility of AI-assisted HRV analysis for early detection and risk stratification. In a retrospective study including 519 participants, ensemble models achieved high classification performance, with CatBoost reaching an accuracy of approximately 91% and an AUC of 0.91, while time-domain and nonlinear HRV indices showed stronger associations with diabetes status than several spectral parameters [120]. However, the cross-sectional design, modest sample size, and potential overfitting of complex models limit the generalizability of these findings, highlighting the need for prospective validation in larger populations.

Another AI model, DiaCardia, demonstrated the feasibility of early prediabetes identification using a standard 12-lead ECG, or even a single electrode, highlighting HRV and R-wave complex changes as non-invasive biomarkers of autonomic dysfunction and subclinical cardiac remodelling. Reduced HRV, observed in both diabetes and metabolic syndrome, suggests that autonomic impairment may precede overt hyperglycaemia, supporting the use of these technologies for non-invasive screening, cardiovascular risk stratification, and guidance of early preventive interventions [117]. However, these findings should be interpreted cautiously because AI-based ECG screening models remain sensitive to differences in acquisition methods, population characteristics, and external validation procedures, so current evidence supports their diagnostic promise more than immediate routine clinical implementation.

Recently, wearable devices have opened new avenues for HRV monitoring in the general population and, in particular, among patients with diabetes, where reduced HRV signals autonomic nervous system dysfunction and increased cardiovascular risk [121]. Studies comparing HRV measurements obtained via wearables with those from clinical ECGs have shown a small absolute error, considered acceptable due to the practicality and low cost of wearable devices, especially for RMSSD and high-frequency bands, although SDNN exhibits a larger error [122,123].

These technologies allow the collection of large longitudinal datasets, which are impossible to obtain under traditional clinical conditions, and their use could enable early identification of autonomic changes and detection of diabetes-associated cardiovascular events [121,124]. However, current limitations include a lack of standardisation between devices, with PPG-based methodologies dominating the market and being sensitive to physical activity and other external factors such as stress, caffeine intake, or hydration [125].

Continuous 24 h monitoring, which captures the cardiovascular response to both diurnal and nocturnal stimuli, remains the gold standard for HRV assessment, but it is difficult to achieve with current wearable devices, making the 24 h Holter ECG the only certified device. In the long term, the development of reliable continuous tracking technologies will be essential for establishing personalised HRV baselines, correlating them with cardiovascular events, and tailoring preventive and therapeutic interventions in patients with diabetes [121].

## 9. Conclusions

HRV analysis in patients with type 2 diabetes demonstrates consistent alterations in cardiac autonomic function, characterised by reductions in several HRV indices, including measures strongly influenced by vagal modulation (e.g., RMSSD, pNN50, HF), together with broader impairment of autonomic cardiovascular regulation. Decreased HRV correlates with the severity of CAN, diabetes duration, glycaemic control, and increased susceptibility to malignant ventricular arrhythmias.

Time-domain, frequency-domain, and nonlinear HRV parameters provide additional insights into electrical instability, serving as a relevant predictive tool for severe cardiovascular events, including sudden cardiac death. Recent studies also suggest that elevated resting heart rate is associated with inadequate glycaemic control and higher HbA1c levels in patients with type 2 diabetes, indicating a potential bidirectional relationship between autonomic function and metabolic regulation, influenced by factors such as BMI. These findings underscore the importance of a personalised approach to patients—not only for individualised clinical management but also for the development of public health strategies aimed at early identification and timely intervention in high-risk populations.

In the long term, the standardised integration of HRV and other autonomic markers, alongside clinical metabolic parameters, into routine practice could become comparable to blood pressure or HbA1c monitoring, providing a practical tool for cardiovascular risk stratification and guidance of personalised interventions. Emerging technologies, including wearable devices and artificial intelligence models, may facilitate continuous monitoring and early detection of autonomic dysfunction, although standardisation and validation of these tools remain essential.

In conclusion, HRV assessment offers unique opportunities for the early detection of CAN, prevention of arrhythmias, and optimisation of cardiovascular management in patients with diabetes, emphasising the importance of personalised medicine and the need for prospective studies to validate interventions and integrate this approach into routine clinical strategies.

## Figures and Tables

**Table 1:** Key HRV Parameters in Patients with Type 2 Diabetes Mellitus and Their Clinical Relevance [22,44,54,55,56,57,58].

Type of Parameter	HRV Parameter	Unit	Typical Alteration in T2DM	Clinical/Prognostic Significance
Time-domain	SDNN	ms	↓ progressively	Loss of cardiac adaptability, with increased risk of mortality and arrhythmias
	RMSSD	ms	↓ early	Reduced parasympathetic tone, with increased risk of atrial fibrillation and ventricular arrhythmias
	pNN50	%	↓	Reduced short-term vagally mediated variability
	SDANN	ms	↓	Loss of 24 h global variability
	SDNN index	ms	↓	5 min segment monitoring for early dysfunction detection
Frequency-domain	VLF power	ms^2^	↓	Independent predictor of cardiovascular mortality
	LF power	ms^2^	↓ or variable	Reflects mixed autonomic and baroreflex modulation rather than a pure marker of sympathetic activity
	HF power	ms^2^	↓	Reduced vagal activity
	LF/HF	%	variable	Composite HRV index influenced by baroreflex and other regulatory mechanisms; it should not be interpreted as a direct measure of sympathovagal balance
	TP (total power)	ms^2^	↓	Reduced global variability and impaired adaptability
	ULF power	ms^2^	↓	Associated with increased risk of mortality and nocturnal arrhythmias
Nonlinear/Linear	SD1	ms	↓	Loss of parasympathetic tone
	SD2	ms	↑	Sympathetic overcompensation
	SD1/SD2	%	↓	Altered autonomic modulation

HRV = heart rate variability; SDNN = standard deviation of NN intervals; RMSSD = root mean square of successive differences; pNN50 = proportion of NN intervals differing by >50 ms; SDANN = standard deviation of average NN intervals over 5 min segments; SDNN index = mean of SDNNs for all 5 min segments; VLF = very low frequency power; LF = low frequency power; HF = high frequency power; LF/HF = ratio of low to high frequency power; TP = total power; ULF = ultra-low frequency power; SD1 = standard deviation perpendicular to line-of-identity (Poincaré); SD2 = standard deviation along line-of-identity (Poincaré); SD1/SD2 = ratio SD1 to SD2. ↓—Down, ↑—Up.

## Data Availability

Not applicable.

## Abbreviations

AF	Atrial fibrillation
AGEs	Advanced glycation end products
BMI	Body mass index
CAN	Cardiac autonomic neuropathy
CI	Confidence interval
CN	Cardiac neuropathy
CV	Cardiovascular
CVR–R	Coefficient of variation of R–R intervals
HF	High-frequency component
HERG	Human ether-à-go-go-related gene
HR	Heart rate
HRV	Heart rate variability
ICD	Implantable Cardioverter-Defibrillator
iNOS	Inducible nitric oxide synthase
Ikr	Rapid delayed rectifier potassium current
LF	Low-frequency component
LF/HF	Ratio of low- to high-frequency power
NN50	Number of NN intervals differing by more than 50 ms
OR	Odds ratio
pNN50	Proportion of NN intervals differing by more than 50 ms
PPG	Photoplethysmography
QTc	Corrected QT interval
RMSSD	Root mean square of successive differences
RAGE	Receptor for advanced glycation end products
ROS	Reactive oxygen species
SD1	Standard deviation perpendicular to line-of-identity (Poincaré)
SD1/SD2	Ratio of SD1 to SD2
SD2	Standard deviation along line-of-identity (Poincaré)
SDANN	Standard deviation of average NN intervals over 5 min segments
SDNN	Standard deviation of NN intervals
SDNN index	Mean of SDNNs for all 5 min segments
SGLT2	Sodium-glucose co-transporter 2 inhibitor
TINN	Triangular interpolation of NN interval histogram
TP	Total power
ULF	Ultra-low frequency power
VLF	Very low frequency power
sDFA	Short-term detrended fluctuation analysis
HbA1c	Glycated haemoglobin
